# Pulmonary thromboendarterectomy for pulmonary hypertension linked to thalassemia

**DOI:** 10.1002/ccr3.3847

**Published:** 2021-01-26

**Authors:** Mohammad Bashar Izzat, Hazem Aljasem, Ahmad Walid Izzat

**Affiliations:** ^1^ Department of Surgery Damascus University Faculty of Medicine Damascus Syria

**Keywords:** cardiopulmonary bypass, heart, hypertension, pulmonary, surgery, thalassemia

## Abstract

This report supports the feasibility of pulmonary thromboendarterectomy in thalassemic patients, and highlights the need for a comprehensive evaluation of the cause of pulmonary hypertension prior to determining the likelihood of surgical cure.

## Graphical abstract

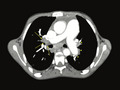

## 1 INTRODUCTION

Pulmonary arterial hypertension (PAH) is a possible complication and a significant source of morbidity and mortality in patients with thalassemia syndromes.^1^ Pathogenic mechanisms of PAH in thalassemia are multifactorial. Chronic hemolysis, reduced nitric oxide bioavailability, chronic hypercoagulable states, and various disease‐directed therapies may play important roles in its development.^2^ Even though chronic thromboembolic pulmonary hypertension (CTEPH) is a well‐recognized cause of PAH in patients with β‐thalassemia, surgical management of CTEPH has only rarely been reported in such cases thus far.^3^, ^4^ We report here the case of a patient with β‐thalassemia complicated with CTEPH who underwent successful surgical pulmonary thromboendarterectomy (PTE). Approval for writing this report was obtained from the local ethics committee of our institution, and obtaining individual consent was waived due to the retrospective nature of the study.

## 2 CASE REPORT

This is the case of a 21‐years‐old man who was diagnosed with β‐thalassemia major at the age of 8 months. He underwent splenectomy at the age of 10 years and has been receiving monthly blood transfusions ever since. Three months prior to admission, he presented to a peripheral emergency department with a sudden episode of dyspnea and central cyanosis and was suspected to be infected with COVID‐19. Chest CT scan was made and ruled out any interstitial lung involvement and revealed the presence of bilateral pulmonary emboli. Anticoagulation therapy with warfarin was started, but on follow‐up the patient developed progressive exertional dyspnea, deteriorating to NYHA functional class III.

**FIGURE 1:**
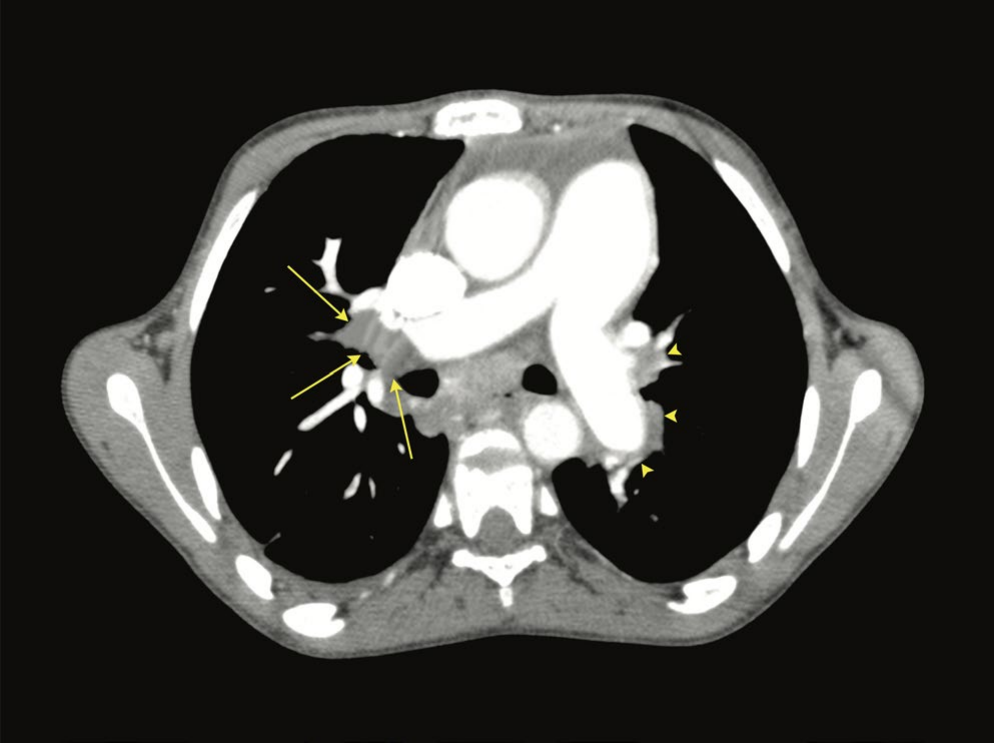
Chest CT angiography showing defects in the right (long arrows) and left (arrow heads) pulmonary arteries in keeping with bilateral chronic pulmonary thromboemboli

The patient was referred to our institution because of worsening symptoms. Physical examination showed clinical evidence of right heart failure and pulmonary hypertension, with a mild drop in arterial oxygen saturation (93%). Echocardiography revealed a dilated right atrium (RA) which contained a large 47 × 35 × 16 mm hyperechogenic mobile mass attached to the RA free wall near the orifice of the inferior vena cava. The right ventricle (RV) was severely dilated and dysfunctional, with tricuspid annular plane systolic excursion (TAPSE) of 13 mm and severe tricuspid regurgitation. Estimated systolic pulmonary arterial pressure (PAP) was 85 mm Hg. Lung perfusion scintigraphy was not available at our center. Chest CT angiography showed bilateral defects in the pulmonary arteries consistent with bilateral chronic pulmonary thromboemboli (Figure 1).

On the basis of this evaluation, the diagnosis of CTEPH was established and PTE was performed, the technique of which was reported previously.^5^ Total circulatory arrest time was 45 minutes, and total cardiopulmonary bypass perfusion time was 94 minutes. A large organized clot was removed from the RA, in addition to removing the thickened intima and organized thrombi from lobar and segmental pulmonary arteries of the left middle and lower lobes, as well as from all major branches on the right.

Early recovery following surgery was uneventful, and the patient was discharged from ICU on the 4th postoperative day. Predischarge echocardiogram confirmed satisfactory hemodynamic results, with an improved RV function and an estimated PAP of 45 mm Hg. The patient was discharged home 19 days after surgery on warfarin but no requirement for supplemental oxygen. Three months after discharge, significant improvements in clinical status (NYHA class I) and oxygen saturation (99%) were noted despite a degree of residual pulmonary hypertension (PAP of 42 mm Hg).

## 3 COMMENT

Valuable advances have been made in understanding the risk factors and mechanisms responsible for the development of PAH in thalassemic patients. It is now known that chronic hemolysis with the resulting nitric oxide depletion can lead to endothelial dysfunction and vascular remodeling and, in due course, to PAH.^1^ Additionally, vascular thromboembolic occlusions can also develop due to in situ thrombosis as well as to pulmonary thromboembolism attributable to the well‐recognized hypercoagulable state.^2^ The relative contribution of hemolysis‐related vasculopathy versus pulmonary thromboembolism in the development of PAH in a specific patient depends on the severity of the genetic defect and the provided treatment methods (such as transfusion rates, chelation therapy, and splenectomy).^1^ This needs to be evaluated in every case individually employing a detailed and systematic analysis of lung perfusion.^6^

Conventional management approaches to PAH in thalassemic patients include hemoglobinopathy‐targeted therapy, PAH‐specific medications as well as general supportive treatment. Existing evidence indicates that currently available drug therapies only temporarily and sporadically improve symptoms in CTEPH and remain ineffective in ameliorating the mechanical obstructions of the pulmonary arteries or in improving patients' survival.^7^ In this context, given that PTE has been established as the gold‐standard therapy for CTEPH, it is conceivable that PTE may comprise the only potentially curative therapy for CTEPH in thalassemic patients.

Experience to date with PTE in thalassemic patients with CTEPH is limited to small series or isolated case reports; nevertheless, these reports favor PTE as an effective treatment of surgically accessible CTEPH.^3^, ^4^ We reported here the successful performance of PTE surgery in a thalassemic patient with CTEPH irresponsive medical treatment. Preoperative assessment indicated that this patient was an appropriate candidate for PTE despite a slightly increased potential surgical risk owing to the underlying thalassemia. The postoperative course was relatively uneventful, and was coupled with an early substantial improvement in hemodynamics and functional status.

It is notable that the postoperative reduction in PAP in our patient was less than that commonly expected following PTE,^3^ which is likely to be due to the multifactorial nature of PAH in thalassemia. In fact, concern remains that the chronic hemolysis‐related vasculopathy in the distal vessels may place thalassemic patients at increased risk of residual or recurrent PAH after PTE.^4^ Therefore, long‐term results of PTE in thalassemic patients remain to be elucidated through long‐term follow‐up. It is noteworthy here that balloon pulmonary angioplasty has recently been proposed as a potential new therapeutic option for this process.^8^

In conclusion, this case report supports the feasibility of PTE in thalassemic patients with surgically accessible CTEPH. Given the complexity of the pathophysiology of PAH in thalassemia, a comprehensive evaluation for both the presence and cause of PAH is imperative prior to determining the likelihood of surgical cure in each individual patient.

## CONFLICT OF INTEREST

The author has declared that no conflict of interest exists.

## AUTHOR CONTRIBUTIONS

MBI: contributed to concept and study design, data interpretation, and writing the manuscript. HA: contributed to data interpretation and approval of manuscript. AWI: contributed to data interpretation and approval of manuscript.

## ETHICAL APPROVAL

All procedures performed in this study were in accordance with the ethical standards of the Damascus University Research Ethics Committee and with the 1964 Helsinki declaration and its later amendments.

## INFORMED CONSENT

Informed consent was obtained from the participant included in the study.

## Data Availability

The data that support the findings of this study are available from the corresponding author, [M. B. I], upon reasonable request.
